# CT-based quantification of intratumoral heterogeneity for predicting pathologic complete response to neoadjuvant immunochemotherapy in non-small cell lung cancer

**DOI:** 10.3389/fimmu.2024.1414954

**Published:** 2024-06-12

**Authors:** Guanchao Ye, Guangyao Wu, Chunyang Zhang, Mingliang Wang, Hong Liu, Enmin Song, Yuzhou Zhuang, Kuo Li, Yu Qi, Yongde Liao

**Affiliations:** ^1^ Department of Thoracic Surgery, Union Hospital, Tongji Medical College, Huazhong University of Science and Technology, Wuhan, China; ^2^ Department of Thoracic Surgery, the First Affiliated Hospital of Zhengzhou University, Zhengzhou University, Zhengzhou, China; ^3^ Department of Radiology, Union Hospital, Tongji Medical College, Huazhong University of Science and Technology, Wuhan, China; ^4^ Department of Thoracic Surgery, Henan Provincial People’s Hospital, Zhengzhou University, Zhengzhou, China; ^5^ School of Computer Science and Technology, Huazhong University of Science and Technology, Wuhan, China

**Keywords:** non-small cell lung cancer, neoadjuvant immunochemotherapy, pathological complete response, radiomics, intratumoral heterogeneity

## Abstract

**Objectives:**

To investigate the prediction of pathologic complete response (pCR) in patients with non-small cell lung cancer (NSCLC) undergoing neoadjuvant immunochemotherapy (NAIC) using quantification of intratumoral heterogeneity from pre-treatment CT image.

**Methods:**

This retrospective study included 178 patients with NSCLC who underwent NAIC at 4 different centers. The training set comprised 108 patients from center A, while the external validation set consisted of 70 patients from center B, center C, and center D. The traditional radiomics model was contrasted using radiomics features. The radiomics features of each pixel within the tumor region of interest (ROI) were extracted. The optimal division of tumor subregions was determined using the K-means unsupervised clustering method. The internal tumor heterogeneity habitat model was developed using the habitats features from each tumor sub-region. The LR algorithm was employed in this study to construct a machine learning prediction model. The diagnostic performance of the model was evaluated using criteria such as area under the receiver operating characteristic curve (AUC), accuracy, specificity, sensitivity, positive predictive value (PPV), and negative predictive value (NPV).

**Results:**

In the training cohort, the traditional radiomics model achieved an AUC of 0.778 [95% confidence interval (CI): 0.688-0.868], while the tumor internal heterogeneity habitat model achieved an AUC of 0.861 (95% CI: 0.789-0.932). The tumor internal heterogeneity habitat model exhibits a higher AUC value. It demonstrates an accuracy of 0.815, surpassing the accuracy of 0.685 achieved by traditional radiomics models. In the external validation cohort, the AUC values of the two models were 0.723 (CI: 0.591-0.855) and 0.781 (95% CI: 0.673-0.889), respectively. The habitat model continues to exhibit higher AUC values. In terms of accuracy evaluation, the tumor heterogeneity habitat model outperforms the traditional radiomics model, achieving a score of 0.743 compared to 0.686.

**Conclusion:**

The quantitative analysis of intratumoral heterogeneity using CT to predict pCR in NSCLC patients undergoing NAIC holds the potential to inform clinical decision-making for resectable NSCLC patients, prevent overtreatment, and enable personalized and precise cancer management.

## Introduction

Neoadjuvant immunochemotherapy, a novel therapeutic approach extensively employed in clinical settings, has demonstrated significant improvements in progression-free survival (PFS) and overall survival (OS) among patients with non small cell lung cancer (NSCLC) (1). With the growing efficacy of neoadjuvant Immunochemistry (NAIC) in the treatment of lung cancer, there has been an increase in the proportion of patients achieving pathological complete response (pCR) (2–4). The proportion of residual tumor cells after surgery is prognostically relevant, with lower residual proportions indicating better prognoses (5). Studies have shown that patients treated with immunotherapy typically achieve good long-term survival outcomes without requiring surgery (6), and the “wait-and-see” approach, which avoids surgical organ preservation, proves to be an effective management choice (7). While this patient group may have achieved pCR, confirmation can only come through histopathological examination of surgically excised specimens. Therefore, the development of a non-invasive and effective method to safely and accurately identify pCR in patients after NAIC remains a significant challenge.

The advancement of artificial intelligence technology has led to the widespread application of machine learning and deep learning across various fields. Radiomics, as a burgeoning method for medical image analysis, employs medical image data to extract numerous quantitative features (8). These features are then analyzed in conjunction with disease characteristics, treatment responses, and patient prognosis (9). Some studies have investigated the predictive value of radiomics features and deep learning radiomics features for NAIC in lung cancer (10–12). Despite the demonstrated performance of traditional radiomics and deep learning radiomics models in predicting the effectiveness of NAIC for lung cancer, these models are unable to capture the spatial heterogeneity of tumors. Typically, quantitative boundary, shape, and texture features are extracted from the tuition region of interest (ROI). This feature extraction method is based on the assumption of uniform tumor heterogeneity. However, in enhanced CT or MR images, changes in the tumor’s internal perfusion give rise to distinct subregions within the tumor, each representing different microstructures (13, 14). Analyzing the subregions of tumors allows for a more accurate representation of their spatial heterogeneity and a realistic restoration of their intrinsic characteristics. Previous research and findings demonstrated that quantitative analysis of MRI heterogeneity in breast cancer prior to treatment can predict the efficacy of neoadjuvant chemotherapy (15). However, quantitative heterogeneity analysis within tumors has not been explored in the context of NAIC for NSCLC.

Thus, the objective of this study is to perform radiomics analysis of quantification of intratumoral heterogeneity in patients with NICA for NSCLC using CT. We establish a quantitative measurement method for tumor heterogeneity and assess whether comparing this method with traditional radiomics model can improve the accuracy of postoperative pCR prediction in patients with NAIC. 

## Materials and methods

### Study sample

This study has received ethical approval from the Ethics Committee of Tongji Medical College Affiliated Union Hospital of Huazhong University of Science and Technology (approval number UCT240116) as well as the Ethics Committees of the participating institutions. As this was a retrospective study, the ethics committee granted exemption from the requirement for informed consent. The study conducted a retrospective screening of clinical and CT scans data from non-small cell lung cancer patients who underwent neoadjuvant immunochemotherapy at Tongji Medical College Affiliated Union Hospital of Huazhong University of Science and Technology (Center A), Zhengzhou University First Affiliated Hospital (Center B), Yichang Central Hospital (Center C), and Anyang Cancer Hospital (Center D). It was registered and accessible on the clinical trials website (https://www.clinicaltrials.gov/) under the registration number NCT06285058.

Inclusion criteria for the study included patients who met the following criteria: (1) Diagnosis of NSCLC confirmed through biopsy pathology and clinical classification as stage IB to III; (2) Received a minimum of two cycles of neoadjuvant immunotherapy in combination with chemotherapy induction therapy; (3) Underwent postoperative pathological evaluation of treatment response in the primary tumor lesion and lymph nodes following the International Association for the Study of Lung Cancer (IASLC) guidelines. Exclusion criteria for the study involved patients who met the following criteria: (1) Absence of contrast enhanced CT image; (2) Incomplete or thick slice CT images of the chest; (3) Interval exceeding one month between the chest CT imaging examination before treatment and treatment initiation; (4) Incomplete or missing clinical pathological data.

**Figure 1:**
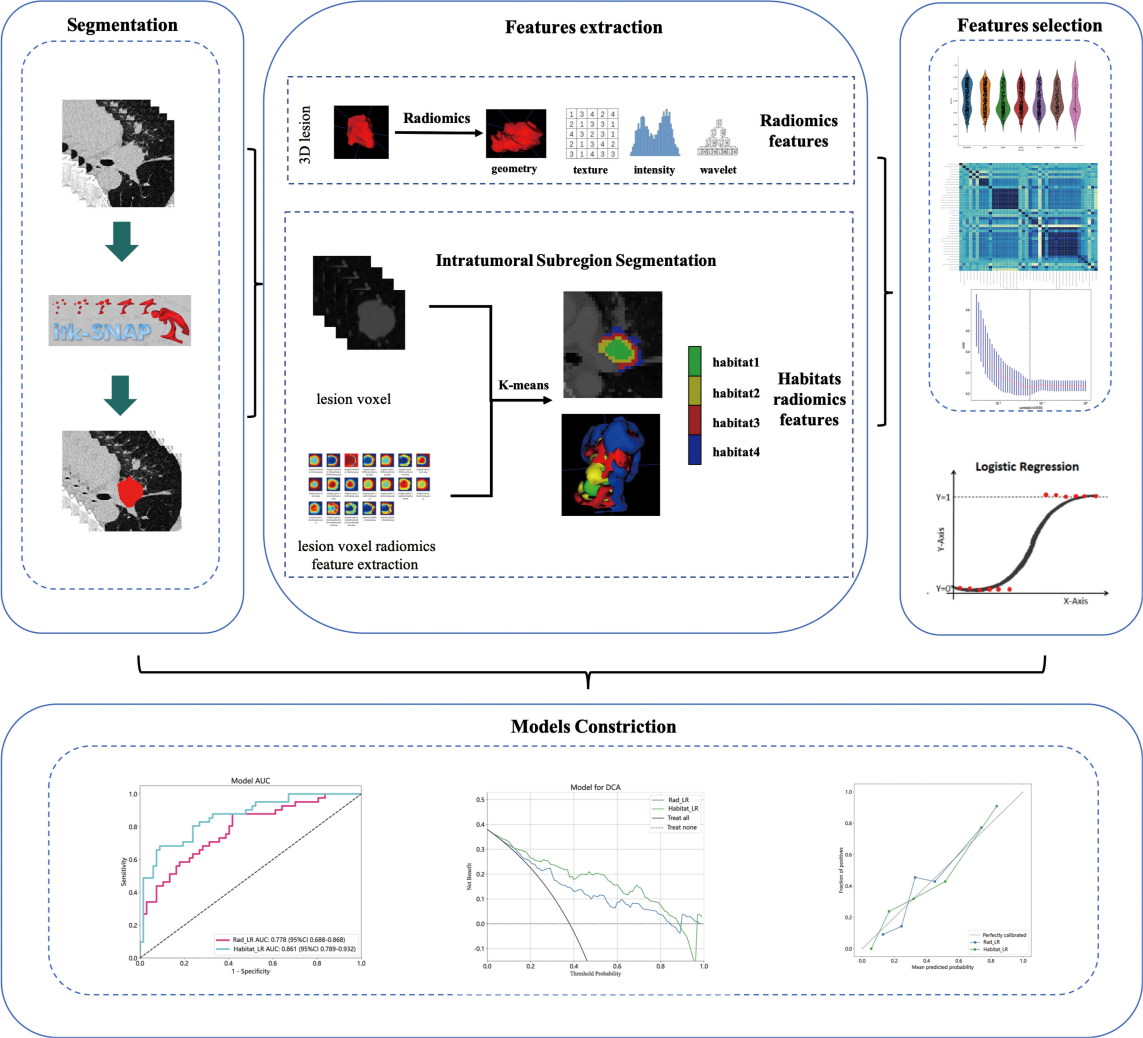
An illustration of the overall research design.

The patient’s clinical and pathological data include factors such as age, gender, smoking history, tumor history, family history, pre-treatment clinical staging, tumor location, pathological type, and postoperative pathological response. Staging was performed using the 8th edition of the TNM staging system by the IASLC (16). A flowchart, as depicted in **Figure 1**, was utilized in this study. This study adhered to the Transparent Reporting of a Multivariate Prediction Model for Individual Prognosis or Diagnosis (TRIPOD) guidelines (17).

### Pretreatment evaluation and neoadjuvant administration

Prior to initiating neoadjuvant therapy, a thorough evaluation of the patient’s diagnosis and staging is necessary, which primarily involves chest-enhanced CT, abdominal-enhanced CT or ultrasound, whole-body bone scintigraphy, brain-enhanced MRI, and positron emission tomography-CT (PET-CT), among others. Pathological diagnosis should be performed using techniques such as bronchoscopy, ultrasound bronchoscopy, and CT-guided percutaneous lung puncture. Immunotherapy drugs commonly used include pembrolizumab, nivolumab, sintilizumab, camrelizumab, tislelizumab, and durvalumab, among others. Chemotherapy primarily involves platinum-based standard dual-drug regimens, administered in cycles every 3 weeks for a treatment duration of 2-4 cycles. The treatment plan, treatment cycle, and surgical timing for the patient are determined by the multidisciplinary team (MDT) based on the patient’s treatment response and overall condition.

### Histopathologic assessment and definition of pCR

In accordance with the IASLC guidelines, proficient pathologists assessed the postoperative pathological response of the primary tumor and lymph nodes in NSCLC patients who underwent NAIC. pCR is defined as the absence of viable tumor cells (ypT0 and ypN0) in both the tumor bed and lymph nodes.

### CT acquisition and preprocessing

**Supplementary S1** provides comprehensive details regarding the acquisition and reconstruction parameters of CT scans conducted at four different centers. Non-ionic iodine contrast agent (350mg/ml) was administered to all patients during the scanning process, with an injection volume ranging from 60 to 80 milliliters and an injection rate of 2 to 3 milliliters per second. The bone reconstruction algorithm is employed for the purpose of reconstruction. Patients are instructed to hold their breath after taking a deep inhalation in a supine position while scanning the area from the lung apex to the level of bilateral costophrenic angles. Finally, arterial phase chest enhanced CT images in digital imaging and communications in medicine (DICOM) format are downloaded from picture archiving and communication systems (PACS). Manual segmentation of lesions is conducted using ITK-SNAP (version 3.8.0, 0) by a radiologist with 10 years of experience and a thoracic surgeon with 5 years of experience. The segmented tumors are jointly verified by senior radiologists and thoracic surgeons. In case of controversial situations, a consensus will be reached through discussions involving four doctors. To assess robustness, a random selection of 50 cases was made to estimate the intra-group correlation coefficients (ICCs), where a value of ≥ 0.75 indicates robustness.

### Radiomics feature extraction and lesion voxel radiomics feature extraction

Extracting radiomics features from ROI using PyRadiomics (http://pyradiomics.readthedocs.io). Radiomics features are divided into three groups: (I) geometric shape, (II) intensity, and (III) texture. Geometric features describe the three-dimensional shape characteristics of tumors. The intensity feature describes the first-order statistical distribution of voxel intensity within tumors. Texture features describe patterns or second-order and higher-order spatial distributions of intensity. For a comprehensive list and explanation of the extracted radiomics features, please refer to **Supplementary S2**. Resample CT images and segmented ROI, and use PyRadiomics to extract radiomics features of each pixel within the tumor ROI. Please refer to **Supplementary S3** for the extracted voxel radiomics features. As shown in **Supplementary S4**, different radiomics features are visualized at each pixel of lesion ROI.

### Intratumoral subregion segmentation and subregional feature extraction

**Figure 2:**
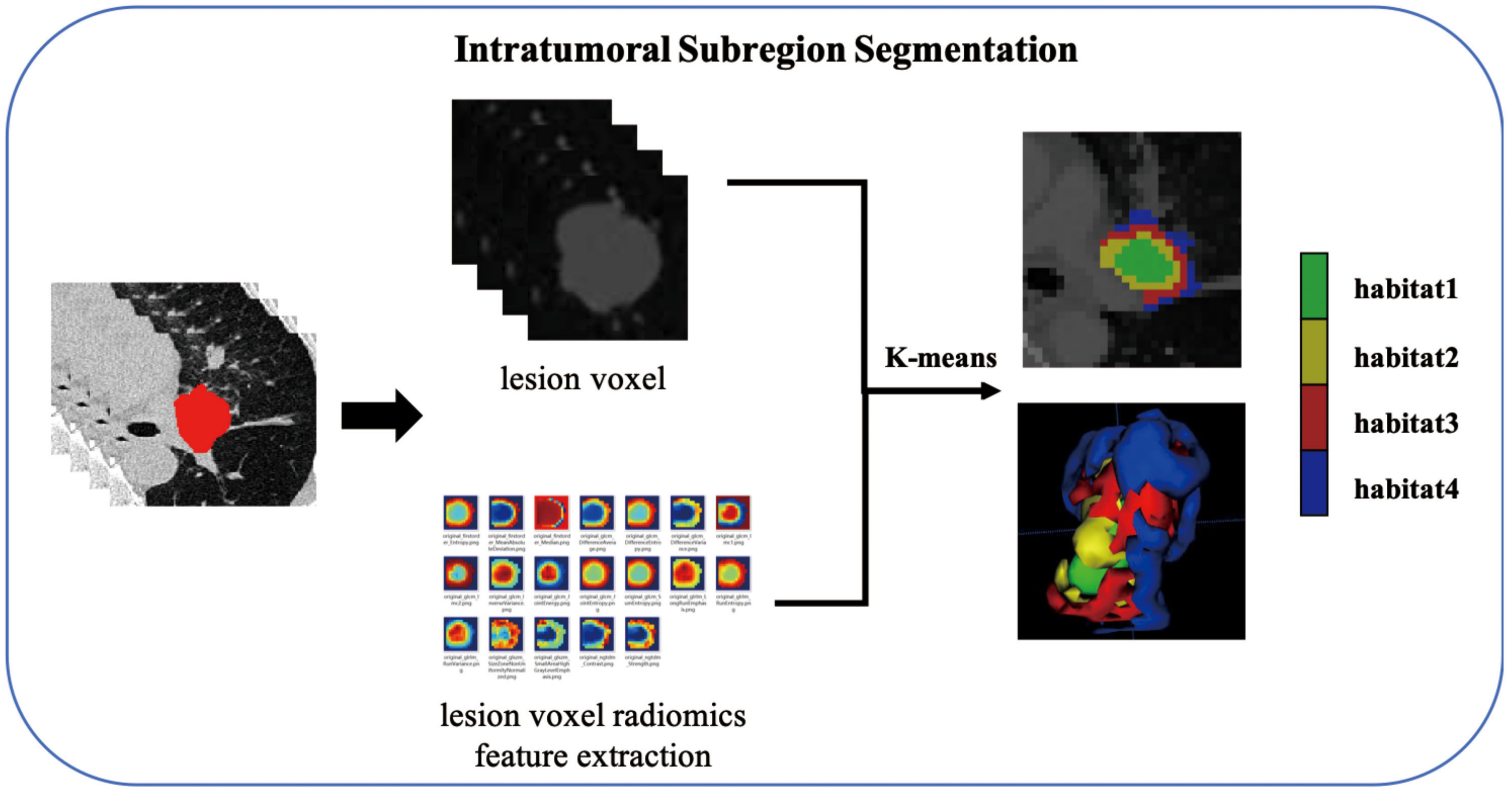
Flow chart of intratumoral Subregion Segmentation.

As shown in the **Figure 2**, the K-Means clustering algorithm is used to cluster the radiomics features of each pixel in the tumor ROI. Use the Calinski-Harabas index (CH) to determine the optimal number of sub regional divisions. Calculate the value of the CH for each variable with K values ranging from 2 to 10. The elbow rule determines the optimal number of clusters by plotting a contour curve of the system clustering analysis. As the number of clusters increases from 2 to 10, the contour value significantly decreases from 4. The optimal number of clusters, which is K=4, corresponds to the turning point in the curve. The best results are attained with 4 clusters, as demonstrated in **Supplementary S5**. **Supplementary S6** provides a visualization depicting the number of clusters in a pCR patient.

Extracting radiomics features from clustered tumor subregions using PyRadiomics (http://pyradiomics.readthedocs.io). Since tumor lesions undergo subregional segmentation, shape features are no longer applicable, and radiomics features are categorized into intensity and texture. The extracted features are divided into: first-order statistics (n=360), GLCM (n=440), GLRLM (n=320), GLSZM (n=320), NGTDM (n=100), and GLDM (n=280). We extract the above radiomics features from each subregion, and the radiomics features extracted from habitat 1 are named feature_h1, and so on.

### Features selection and prediction model constriction

To ensure balanced data, minimize differences resulting from feature distribution, prevent overfitting, and enhance the model’s generalization ability, Z-score regularization was initially employed to standardize the extracted radiomics data to have a mean of 0 and a variance of 1. Features selection was conducted using a *t*-test, and features with a *p*-value less than 0.05 were further analyzed. The Pearson correlation coefficient was used to filter features, retaining only one of two features if their correlation coefficient exceeded 0.9. Regression feature selection was performed using the Least Absolute Shrinkage and Selection Operator (LASSO) algorithm, and cross-validation was used to determine the weights of features for different Lambda values. The penalty coefficient obtained from cross-validation served as the basis for model training, and features with a coefficient greater than 0 were filtered out, with the corresponding formula printed.

PyRadiomics is utilized to extract the radiomics features of each subregion, based on the optimal number of clusters. The radiomics features from each subregion are fused and incorporated into the LR algorithm to construct a prediction model for analyzing intratumoral heterogeneity.

### Model evaluation and statistical analysis

The diagnostic performance of the model was evaluated using criteria such as AUC, accuracy, specificity, sensitivity, PPV, and NPV. The decision curve analysis (DCA) evaluation model was employed to predict the net benefit of NAIC efficacy. Additionally, the model calibration curve was utilized to demonstrate the comparative effect between the constructed prediction model and the perfect fit. The Hosmer Lemeshow test was used to determine the difference between predicted and true values.

Statistical analysis was conducted using SPSS software (version 27.0, IBM) and Python software (version 3.5.6; http://www.python.org). The independent sample *t*-test was performed to analyze continuous variables, expressed as mean ± standard deviation. The correlation between categorical variables was analyzed using the chi-square test or Fisher’s exact test, and the results were represented as a ratio. Pearson correlation test was employed to analyze the correlation between features and avoid highly repetitive features. If the correlation coefficient between any two features exceeds 0.9, only one feature is retained. LASSO regression was utilized to select non-zero variables as important features and incorporate them into the prediction model. The significance level was set at *p*<0.05.

## Results

### Patient characteristics

**Figure 3:**
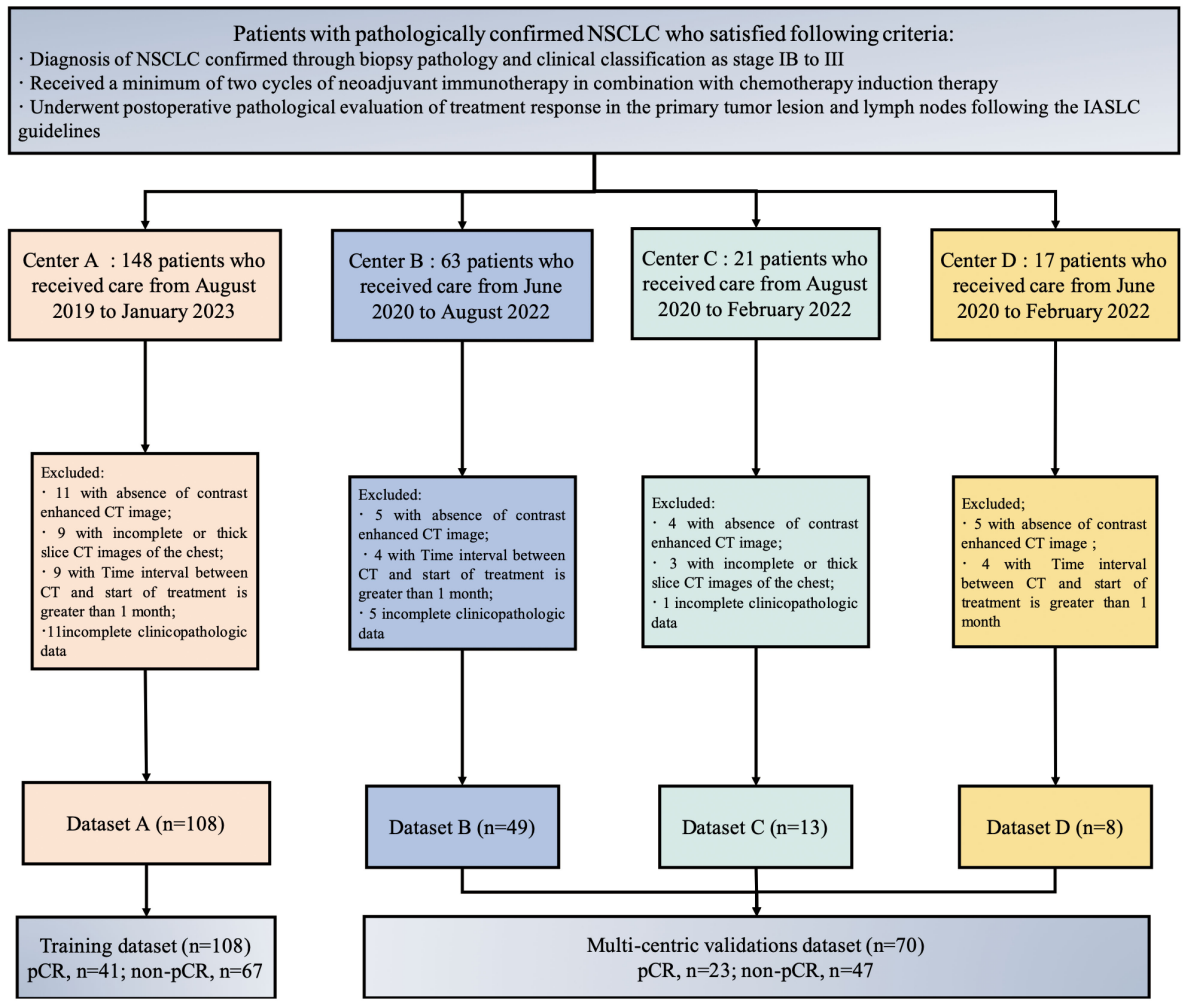
Flowchart shows patient exclusion for each dataset.

A total of 250 patients who underwent NAIC were screened in four hospitals, based on the inclusion criteria. Patients were excluded based on the following criteria: 26 patients who did not undergo pre-treatment chest enhanced CT examination, 15 patients with incomplete CT images or who underwent thick slice scanning; 12 patients who had chest CT imaging examinations before treatment with a time interval of more than 1 month between the start of treatment, and 19 patients with incomplete clinical and pathological data or other missing information. In total, 178 patients were included in the study, comprising 108 patients from Center A, 49 from Center B, 13 from Center C, and 8 from Center D. The training set consisted of 108 patients from Center A, while the external validation set comprised 70 patients from Center B, Center C, and Center D, as illustrated in **Figure 3**.

**Table 1** presents the general statistical analysis of patient data. Out of the patients who received NAIC, 64 achieved pCR, while 114 did not, resulting in a pCR rate of 36.0%. The study included 155 male cases (87.1%) and 23 female cases (12.9%). The study identified statistically significant differences (*p* < 0.05 for all) in various factors, including gender, family history and T stage. Conversely, no statistically significant differences were observed between the two groups in terms of smoking, tumor history, location of lesion, pathological type, N stage and clinical stage. **Table 2** demonstrates that the distribution of clinicopathological characteristics is similar in both the training cohort and external validation cohort.

**Table 1:** Comparison of clinical and pathological characteristics between pCR group and non- pCR group.

Characteristics	Total (n=178)	Non-pCR group (n=114)	pCR group (n=64)	*p* value
Age(years), Mean ±SD	60.243±8.079	60.492±7.554	59.783±8.982	0.575
Gender, n (%)				0.007
Female	23(12.9)	21(18.4)	2(3.1)	
Male	155(87.1)	93(81.6)	62(96.9)	
Smoking, n (%)				0.153
Yes	110(61.8)	66(57.9)	44(68.8)	
No	68(38.2)	48(42.1)	20(31.2)	
Family history, n (%)				0.016
Yes	4(2.2)	0(0.0)	4(6.2)	
No	174(97.8)	114(100.0)	60(93.8)	
Tumor history, n (%)				1.000
Yes	2(1.1)	1(0.9)	1(1.6)	
No	176(98.9)	113(99.1)	63(98.4)	
Location of lesion, n (%)				0.294
right	88(49.4)	53(46.5)	35(53.7)	
left	90(50.6)	61(53.5)	29(45.3)	
Pathological type, n (%)				0.448
Squamous carcinoma	133(74.7)	82(71.9)	51(79.7)	
adenocarcinoma	40(22.5)	29(25.4)	11(14.4)	
other	5(2.8)	3(2.6)	2(3.1)	
T stage, n (%)				0.003
T1	23(12.9)	7(6.1)	16(25.0)	
T2	64(36.0)	41(36.0)	23(35.9)	
T3	60(33.7)	43(57.7)	17(26.6)	
T4	31(17.4)	23(20.2)	8(12.5)	
N stage, n (%)				0.865
N0	14(7.9)	10(8.8)	4(6.2)	
N1	35(19.6)	23(20.1)	12(18.8)	
N2	116(65.2)	72(63.2)	44(68.8)	
N3	13(7.3)	9(7.9)	4(6.2)	
Clinical stage, n (%)				0.734
II	23(12.9)	14(12.3)	9(14.1)	
III	155(87.1)	100(87.7)	55(85.9)	

pCR, pathological complete response.

**Table 2:** Patient characteristics across different cohorts.

Characteristics	Total (n=178)	Training (n=108)	Validation (n=70)	*p* value
Age(years), Mean ±SD	60.243±8.079	60.193±6.927	60.30±9.643	0.932
Gender, n (%)				0.023
Female	23(12.9)	9(8.3)	14(20.0)	
Male	155 (87.1)	99(91.7)	56(80.0)	
Smoking, n (%)				0.387
Yes	110(61.8)	64(59.3)	46(65.7)	
No	68(38.2)	44(40.7)	24(34.3)	
Family history, n (%)				0.647
Yes	4(2.2)	2(1.9)	2(2.9)	
No	174(97.8)	106(98.1)	68(07.1)	
Tumor history, n (%)				0.153
Yes	2(1.1)	0(0.0)	2(2.9)	
No	176(98.9)	108(100.0)	68(97.1)	
Location of lesion, n (%)				0.424
right	88(49.4)	56(51.9)	32(45.7)	
left	90(50.6)	52(48.1)	38(54.3)	
Pathological type, n (%)				0.605
Squamous carcinoma	133(74.7)	79(73.1)	54(77.1)	
adenocarcinoma	40(22.5)	25(23.1)	15(21.4)	
other	5(2.8)	4(3.7)	1(1.4)	
Clinical T stage, n (%)				0.206
T1	23(12.9)	11(10.2)	12(17.1)	
T2	64(36.0)	45(41.7)	19(27.1)	
T3	60(33.7)	34(31.5)	26(37.1)	
T4	31(17.4)	18(16.6)	13(18.6)	
Clinical N stage, n (%)				0.881
N0	14(7.9)	8(7.5)	6(8.6)	
N1	35(19.7)	20(18.5)	15(21.4)	
N2	116(65.1)	71(65.7)	45(64.3)	
N3	13(7.3)	9(8.3)	4(5.7)	
Clinical stage, n (%)				0.371
II	23(12.9)	12(11.1)	11(15.7)	
III	155(87.1)	96(88.9)	59(84.3)	
pCR, n (%)				0.488
Present	64(36.0)	41(38.0)	23(32.9)	
Absent	114(64.0)	67(62.0)	47(67.1)	

pCR, pathological complete response.

### Construction of radiomics feature model

**Figure 4:**
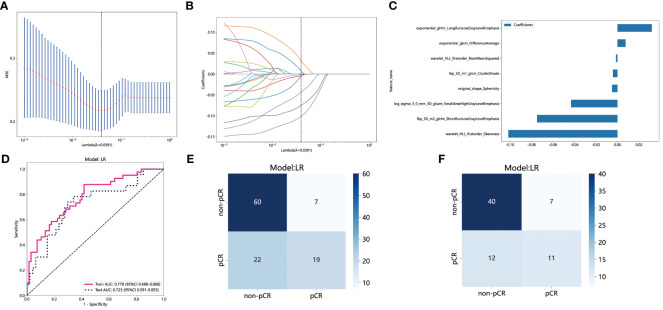
Radiomics feature selection based on LASSO algorithm and construction of the radiomics model. **(A)** the MSE of LASSO regression. **(B)** the coefficients for cross-validation of LASSO regression. **(C)** Selected features weight coefficients. **(D) **The ROC curves of the radiomics model in the training and validation cohorts. **(E)** Confusion matrix for radiomics model in the training cohort. **(F)** Confusion matrix for radiomics model in the external validation cohort. LASSO, least absolute shrinkage and selection operator; MSE, mean standard error; ROC, receiver operating characteristic.

The PyRadiomics package was utilized in this study to extract 1834 features from the ROI for each patient. Following reproducibility evaluation using ICCs, 1326 features were deemed reliable and selected for further analysis. 42 features exhibiting statistical differences between the pCR group and the non-pCR group were selected using a *t*-test. Subsequently, Pearson correlation coefficient analysis was conducted on these features to examine the pairwise correlations among them. Only features with a correlation coefficient greater than 0.9 were retained, selecting one representative feature. Ultimately, 22 features were chosen for subsequent analysis. Employing LASSO regression screening, 8 features with non-zero coefficients were selected from the initial set of 22 features to create a Rad score. **Figure 4A** displays the mean standard error (MSE) of LASSO regression, **Figure 4B** illustrates the coefficients for cross-validation of LASSO regression, and **Figure 4C** presents the coefficient values of the final selected non-zero features. The selected 8 traditional radiomics features were incorporated into the machine learning algorithm, LR, to construct a traditional radiomics model.

As depicted in **Figure 4D**, the AUC of this model was 0.778 (95% CI: 0.688-0.868) in the training cohort and 0.723 (95% CI: 0.591-0.855) in the external validation cohort. **Figures 4E** and **4F** depict the confusion matrix of the traditional radiomics model. In the training set, the traditional radiomics model achieved an accuracy of 0.685, sensitivity of 0.854, specificity of 0.582, PPV of 0.556, and NPV of 0.867. In the external validation set, the model achieved an accuracy of 0.686, sensitivity of 0.739, specificity of 0.660, PPV of 0.515, and NPV of 0.838.

### Construction of habitat model

**Figure 5:**
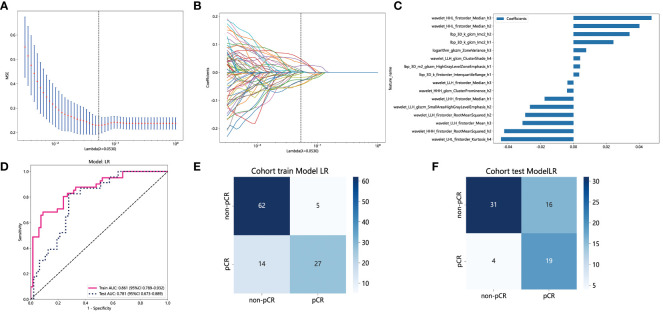
Habitats radiomics feature selection based on LASSO algorithm and construction of the radiomics model. **(A)** the MSE of LASSO regression. **(B)** the coefficients for cross-validation of LASSO regression. **(C)** Selected features weight coefficients. **(D)**The ROC curves of the habitats model in the training and validation cohorts. **(E)** Confusion matrix for habitats model in the training cohort. **(F)** Confusion matrix for habitats model in the external validation cohort. LASSO, least absolute shrinkage and selection operator; MSE, mean standard error; ROC, receiver operating characteristic.

Habitats radiomics features are extracted from four tumor subregions using PyRadiomics. The features extracted from each subregion are collectively referred to as feature_h1, feature_h2, feature_h3, and feature_h4. Habitat 1, Habitat 2, Habitat 3, and Habitat 4 each extract 1820 features. These features are then fused, screened, and used to construct a model. A total of 7280 habitats radiomics features were extracted from the 4 tumor subregions. A total of 221 features exhibiting statistical differences were selected between the pCR group and the non-pCR group using a *t*-test. Subsequently, a Pearson correlation coefficient analysis was conducted on these features, retaining only those with a correlation coefficient greater than 0.9. Finally, 101 features were chosen for further analysis. By employing LASSO regression screening, 16 features with non-zero coefficients were selected from the initial 101 features to establish the tumor’s internal heterogeneity habitat score. **Figure 5A** displays the MSE of LASSO regression, **Figure 5B** illustrates the coefficients obtained through cross-validation in LASSO regression, and **Figure 5C** presents the coefficient values of the final selected non-zero features. The 16 selected radiomics features of the habitat are incorporated into the machine learning algorithm, LR, to construct a model for tumor internal heterogeneity habitat. The AUC of this model was 0.861 (95% CI: 0.789-0.932) in the training group and 0.781 (95% CI: 0.673-0.889) in the external validation group, as depicted in **Figure 5D**. **Figures 5E** and **5F** display the confusion matrix of the tumor internal heterogeneity habitat model. In the training set, the tumor internal heterogeneity habitat model achieved the following performance metrics: accuracy of 0.815, sensitivity of 0.659, specificity of 0.910, PPV of 0.818, and NPV of 0.813. In the external validation queue, the model achieved an accuracy of 0.743, sensitivity of 0.783, specificity of 0.723, PPV of 0.581, and NPV of 0.872.

### Comparison of the radiomics feature model and habitat model

**Figure 6:**
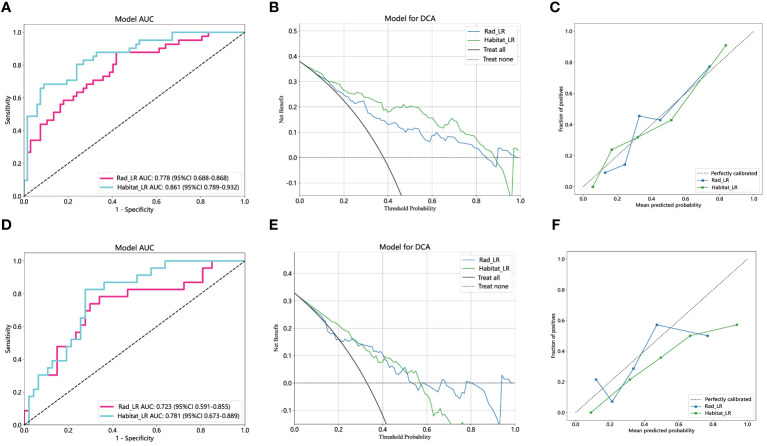
The performance of radiomics model and habitats model in the training and the external validation cohorts. The AUC, DCA and Calibration curves of radiomics model and habitats model in the training cohort **(A, B, C)** and the external validation cohort **(D, E, F)**. AUC, area under the receiver operating characteristic curve; DCA, decision curve analysis.

The performance of the radiomics model and habitats model in the training cohort and external validation cohort is summarized in **Table 3**. **Figure 6A** and **Figure 6D** display the ROC curves and corresponding AUC results of two prediction models, namely the traditional radiomics model and the tumor heterogeneity habitat model, used for predicting pCR in NAIC for NSCLC. The evaluation is performed on both the training and external validation cohorts. **Figures 6B** and **6E** illustrate the decision curves of the two prediction models in the training and validation sets, respectively. The utilization of these two prediction models yields a higher overall net benefit in predicting pCR for patients undergoing NAIC in NSCLC, as compared to non-intervention patients. The calibration curves of the two prediction models, depicted in **Figures 6C** and **6F**, showcase the performance of these models in relation to perfect fitting, across both the training and validation groups. The Hosmer Lemeshow test serves as a model fitting indicator, assessing the disparity between predicted and true values. A *p*-value greater than 0.05 indicates a successful passing of the Hosmer Lemeshow test, implying no significant difference between the predicted and true values. In the training cohort, the p-values of the Hosmer Lemeshow test for the radiomics model and the habitat model are 0.325 and 0.452, respectively. The model fit of the external validation group is slightly inferior when compared to the training group.

**Table 3:** Discrimination performance comparison of the prediction models for pCR.

	AUC	ACC	Sensitivity	Specificity	PPV	NPV
Rad model						
Training	0.778	0.685	0.854	0.582	0.556	0.867
Validation	0.723	0.686	0.739	0.660	0.515	0.838
Habitat model						
Training	0.861	0.815	0.659	0.910	0.818	0.813
Validation	0.781	0.743	0.783	0.723	0.581	0.872

pCR, pathological complete response; AUC, area under curve; ACC, accuracy; PPV, positive predictive value; NPV, negative predictive value.

## Discussion

The study comprised a total of 178 patients diagnosed with NSCLC who underwent NAIC. A model utilizing CT-extracted imaging features prior to treatment was constructed to predict pCR. The results demonstrated that the habitat model, constructed through quantitative analysis of tumor internal heterogeneity, achieved an AUC value of 0.861 in the training group, outperforming the traditional radiomics model with an AUC of 0.778. In the validation group, the tumor internal heterogeneity habitat model exhibited an AUC value of 0.781, surpassing the AUC of the traditional radiomics model at 0.723.

NAIC is an emerging adjuvant therapy for lung cancer primarily aimed at inhibiting tumor immune escape, activating the body’s immune response, and eliminating tumor cells (18). Nevertheless, variations in the efficacy of NAIC exist, and currently, there are no effective screening methods to identify individuals who respond favorably to NAIC. Traditional radiological methods face challenges in evaluating their effectiveness. Radiomics can extract features from CT images of lung cancer, establish a predictive model for assessing the efficacy of NAIC in lung cancer, enable early identification of drug resistance, and offer guidance for optimizing and modifying therapy plans (19). Yang et al. (3) developed a predictive model utilizing radiomics features extracted from CT scans taken before and after NAIC to predict pathological response, achieving an AUC of 0.85 and an accuracy of 81%. Similarly, Han et al. (20) extracted radiomics features from CT data obtained before and after NAIC for lung cancer and constructed a model to predict MPR, achieving a maximum AUC value of 0.850. Liu et al. (10) enrolled 89 patients with lung cancer who received NAIC and incorporated clinical data and radiomics features to build a nomogram model that integrated both types of features. The training set yielded an AUC of 0.84 and an accuracy of 80%. Deep learning, a complex machine learning algorithm, has demonstrated superior performance in language and image recognition compared to preceding technologies. The application of deep learning in medical image processing has garnered growing attention. Deep learning-based models have exhibited satisfactory performance in predicting the primary pathological response to NAIC in patients with NSCLC. Lin et al. (11) conducted a retrospective analysis of clinical and imaging data from 62 patients with NSCLC who underwent NAIC. They extracted radiomics and deep learning features from lung cancer lesions and constructed an integrated model that combines clinical features, radiomics features, and deep learning features to achieve accurate efficacy prediction. She et al. (21) integrated multicenter data and developed a model for predicting MPR in patients undergoing NAIC using deep learning features. In the external validation set, the deep learning model achieved an AUC of 0.72 for MPR prediction. In conclusion, radiomics and deep learning models based on artificial intelligence have demonstrated significant potential in predicting the effectiveness of NAIC for lung cancer. These models can aid physicians in early identification of patient responses and drug resistance, thereby offering valuable insights for optimizing and transitioning immunotherapy treatment plans.

Despite the high predictive performance of traditional radiomics and deep learning radiomics models, these models fail to capture the spatial heterogeneity of tumors in their features. Tumor heterogeneity can be categorized into two types: spatial heterogeneity in the spatial dimension and temporal heterogeneity in the temporal dimension (22). Spatial heterogeneity in tumors pertains to the distinct characteristics and distribution of internal cells and tissues. Tumors are heterogeneous entities consisting of diverse cell types, such as tumor cells, blood vessels, immune cells, and fibrous tissue (23). Such heterogeneity frequently results in irregular tumor growth patterns, along with inconsistent distribution and density of cells. The spatial heterogeneity of tumors profoundly influences tumor treatment. Tumor cells in distinct regions may exhibit varying sensitivities to therapeutic drugs, while the low oxygen and nutrient-deprived areas within the tumor may pose challenges for drug delivery (24). Therefore, it is crucial to understand and consider the spatial heterogeneity of tumors when developing effective treatment strategies. Neoadjuvant therapy patients who are ineligible or temporarily unfit for surgery cannot have the tumor completely removed and evaluate tumor heterogeneity through pathology. The development of artificial intelligence radiomics enables quantitative imaging, which quantifies and analyzes tumor imaging data, thereby obtaining digital information that is more accurate, objective, and reproducible. Analyzing the subregions of tumors enables a better reflection of their spatial heterogeneity and a more realistic restoration of their characteristics. Utilizing this quantitative data to comprehend tumor heterogeneity aids in tumor diagnosis, classification, grading, and evaluation of treatment response, thereby assisting doctors in formulating personalized treatment strategies (25, 26). Tumor heterogeneity plays a crucial role in predicting tumor progression, assessing therapeutic effects, and devising personalized treatment plans (27). This study aimed to analyze the internal microscopic manifestations of tumors using imaging and investigate the spatial manifestations of tumor heterogeneity. Additionally, it introduced a habitat model for analyzing tumor internal heterogeneity to predict pCR in NAIC patients with NSCLC. The K-means unsupervised clustering method was employed to partition tumor subregions, and the habitat model, which extracted subregional radiomics features, notably enhanced the predictive performance of radiomics. The habitat model, constructed using subregional radiomics features, plays a crucial role in predicting the efficacy of NAIC for NSCLC. This model can provide additional tumor information, enhance the objectivity and accuracy of efficacy evaluation, optimize treatment plan selection and adjustment, and improve treatment effectiveness and safety.

This study has several limitations and shortcomings. Firstly, as a retrospective study, it may be prone to selection bias. Additionally, there might be statistical gender differences between the training group and validation group, potentially impacting the model’s accuracy. Future prospective clinical studies are needed to validate the model’s accuracy and enhance its performance. Secondly, although it is a multicenter study, the inclusion of patient data from each center was not substantial. Future research endeavors to accomplish large-scale, multicenter validation. Thirdly, this study solely incorporated pre-treatment CT data and excluded CT data after neoadjuvant therapy. Integrating temporal analysis alongside spatial heterogeneity could enhance the predictive model’s performance. Subsequent research will analyze both spatial and temporal heterogeneity to construct predictive models with enhanced accuracy. Fourthly, this study omitted patient pre-treatment puncture specimen information, such as PD-L1 expression, TMB, and sequencing data. In the future, it is worth considering the incorporation of puncture pathology data and the development of a multimodal prediction model to further enhance the model’s accuracy.

In conclusion, radiomics analysis utilizing CT image was performed to evaluate the intratumoral heterogeneity in NSCLC patients undergoing NAIC treatment. The tumor heterogeneity analysis habitat model, when compared to conventional radiomics models, exhibits enhanced accuracy in predicting postoperative pCR in patients with NAIC. This approach has the potential to aid in clinical decision-making for patients with resectable NSCLC, prevent unnecessary treatment, and facilitate personalized and precise cancer management.

## Data availability statement

The original contributions presented in the study are included in the article/**Supplementary Material**. Further inquiries can be directed to the corresponding author.

## Ethics statement

Written informed consent was not obtained from the individual(s) for the publication of any potentially identifiable images or data included in this article as this was a retrospective study, therefore, the ethics committee granted exemption from the requirement for informed consent.

## Author contributions

GY: Data curation, Methodology, Writing – original draft, Writing – review & editing. GW: Data curation, Methodology, Writing – original draft, Writing – review & editing. CZ: Data curation, Writing – review & editing. MW: Data curation, Writing – review & editing. HL: Software, Writing – review & editing. ES: Software, Writing – review & editing. YZ: Software, Writing – review & editing. KL: Conceptualization, Data curation, Supervision, Writing – review & editing. YQ: Conceptualization, Data curation, Supervision, Writing – review & editing. YL: Writing – review & editing.
