# Supplementary material for: CT-based quantification of intratumoral heterogeneity for predicting pathologic complete response to neoadjuvant immunochemotherapy in non-small cell lung cancer
Source: Front Immunol. 2024 Jun 12;15:1414954. doi: 10.3389/fimmu.2024.1414954 (PMC11199789; doi:10.3389/fimmu.2024.1414954)
Supplement: Supplementary file 1 [file DataSheet_1.docx]

**Supplementary S1: CT scanning parameters among multiple centers.**

| Parameters | Center A | Center B | Center C | Center D |
| --- | --- | --- | --- | --- |
| Manufacturer | Siemens | GE | GE | Siemens |
| Acquisition type | Helical | Helical | Helical | Helical |
| Tube voltage | 120 kVp | 120 kVp | 120 kVp | 120 kVp |
| Tube current | automatically | automatically | automatically | automatically |
| Pitch | 0.75-1 | 0.625-5 | 1.2-1.5 | 1-1.4 |
| Collimation | 0.6 | 0.6 | 0.6 | 0.6 |
| Matrix | 512 × 512 | 512 × 512 | 512 × 512 | 512 × 512 |
| Pixel spacing | 0.75mm | 0.6-0.96 mm | 0.6-0.96 mm | 0.6-1.0mm |
| Reconstructed slice thickness | 0.625mm | 1mm | 0.625mm | 0.625mm |

**Supplementary S2: the extracted features are categorized into seven distinct groups**

| Features | Number |
| --- | --- |
| shape | n=14 |
| grey level co-occurrence matrix (GLCM) | n=440 |
| first-order statistics | n=360 |
| grey level size zone matrix (GLSZM) | n=320 |
| neighborhood gray-tone difference matrix (NGTDM) | n=100 |
| grey level run length matrix (GLRLM) | n=320 |
| gray level dependence matrix (GLDM) | n=280 |

**Supplementary S3: the extracted voxel radiomics features**

| Categorization | Features |
| --- | --- |
| first-order statistics, n=3 | firstorder_Entropy, firstorder_MeanAbsoluteDeviation, firstorder_Median |
| grey level co-occurrence matrix (GLCM), n=9 | glcm_DifferenceAverage, glcm_DifferenceEntropy, glcm_DifferenceVaria, glcm_lmc1, glcm_lmc2, glcm_lnverseVariance, glcm_JointEnergy, glcm_JointEntropy, glcm_SumEntropy |
| grey level run length matrix (GLRLM), n=3 | glrlm_LongRunEmphasis, glrlm_RunEntropy, glrlm_RunVariance |
| grey level size zone matrix (GLSZM), n=2 | glszm_SizeZoneNonUniformityNormalized, glszm_SmallAreaHighGrayLevelEmphasis |
| neighborhood gray-tone difference matrix (NGTDM), n=2 | ngtdm_Contrast, ngtdm_Strength |

**Supplementary S4: different radiomics features are visualized at each pixel of lesion ROI**


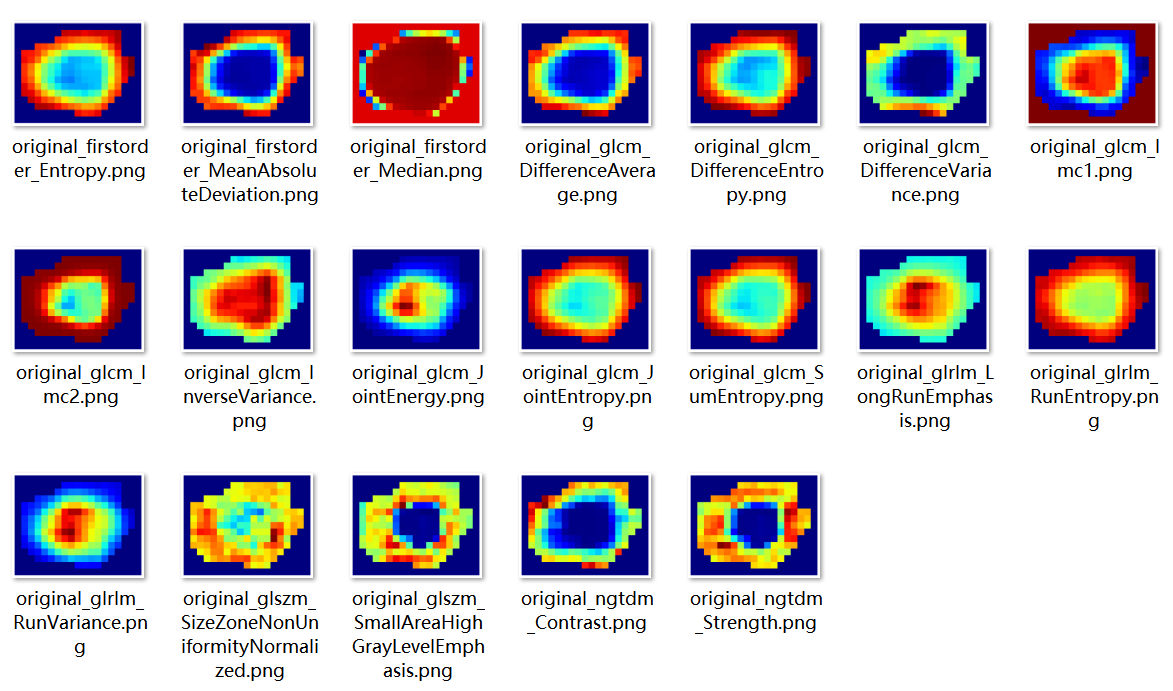


**Supplementary S5: the Calinski-Harabas index (CH) to determine the optimal number of sub regional divisions**

**
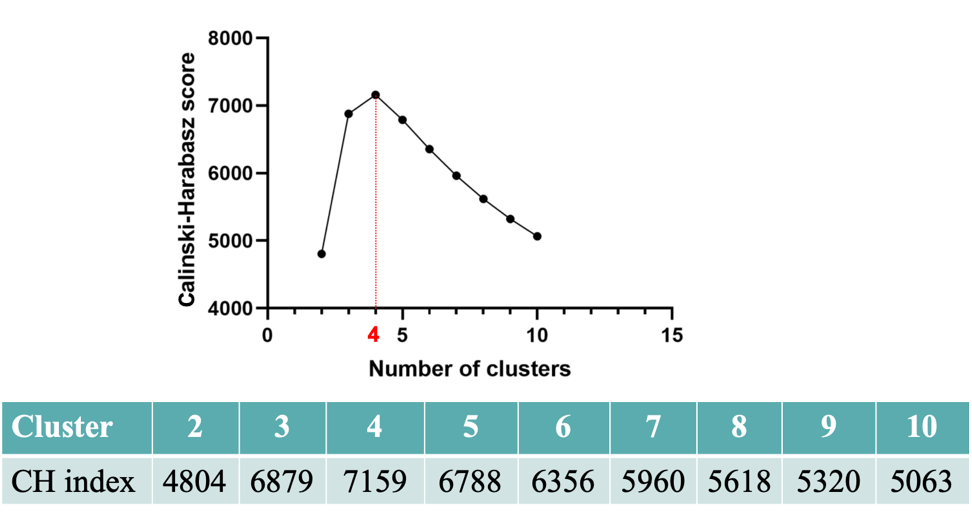
**

**Supplementary S6:** **a visualization depicting the number of clusters in a pCR patient**

**
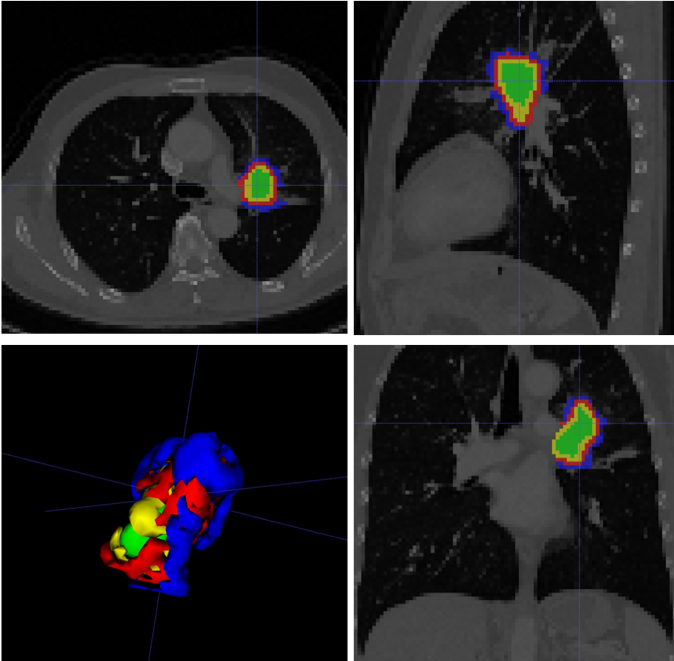
**

**Supplementary S7: TRIPOD checklist**

| **Section/Topic** | **Item** |  | **Checklist Item** | **Page** |
| --- | --- | --- | --- | --- |
| **Title and abstract** | | | | |
| Title | 1 | D;V | Identify the study as developing and/or validating a multivariable prediction model, the target population, and the outcome to be predicted. | 1 |
| Abstract | 2 | D;V | Provide a summary of objectives, study design, setting, participants, sample size, predictors, outcome, statistical analysis, results, and conclusions. | 1-2 |
| **Introduction** | | | | |
| Background and objectives | 3a | D;V | Explain the medical context (including whether diagnostic or prognostic) and rationale for developing or validating the multivariable prediction model, including references to existing models. | 3-4 |
|  | 3b | D;V | Specify the objectives, including whether the study describes the development or validation of the model or both. | 3-4 |
| **Methods** | | | | |
| Source of data | 4a | D;V | Describe the study design or source of data (e.g., randomized trial, cohort, or registry data), separately for the development and validation data sets, if applicable. | 4 |
|  | 4b | D;V | Specify the key study dates, including start of accrual; end of accrual; and, if applicable, end of follow-up. | 4 |
| Participants | 5a | D;V | Specify key elements of the study setting (e.g., primary care, secondary care, general population) including number and location of centres. | 4 |
|  | 5b | D;V | Describe eligibility criteria for participants. | 4-5/Fig 3 |
|  | 5c | D;V | Give details of treatments received, if relevant. | - |
| Outcome | 6a | D;V | Clearly define the outcome that is predicted by the prediction model, including how and when assessed. | 5-6 |
|  | 6b | D;V | Report any actions to blind assessment of the outcome to be predicted. | 5-6 |
| Predictors | 7a | D;V | Clearly define all predictors used in developing the multivariable prediction model, including how and when they were measured. | 6-7 |
|  | 7b | D;V | Report any actions to blind assessment of predictors for the outcome and other predictors. | 6-7 |
| Sample size | 8 | D;V | Explain how the study size was arrived at. | Fig 3 |
| Missing data | 9 | D;V | Describe how missing data were handled (e.g., complete-case analysis, single imputation, multiple imputation) with details of any imputation method. | - |
| Statistical analysis methods | 10a | D | Describe how predictors were handled in the analyses. | 7-8 |
|  | 10b | D | Specify type of model, all model-building procedures (including any predictor selection), and method for internal validation. | 7-8 |
|  | 10c | V | For validation, describe how the predictions were calculated. | 8 |
|  | 10d | D;V | Specify all measures used to assess model performance and, if relevant, to compare multiple models. | 8 |
|  | 10e | V | Describe any model updating (e.g., recalibration) arising from the validation, if done. | - |
| Risk groups | 11 | D;V | Provide details on how risk groups were created, if done. | - |
| Development vs. validation | 12 | V | For validation, identify any differences from the development data in setting, eligibility criteria, outcome, and predictors. | - |
| **Results** | | | | |
| Participants | 13a | D;V | Describe the flow of participants through the study, including the number of participants with and without the outcome and, if applicable, a summary of the follow-up time. A diagram may be helpful. | Fig 3 |
|  | 13b | D;V | Describe the characteristics of the participants (basic demographics, clinical features, available predictors), including the number of participants with missing data for predictors and outcome. | 9/Table 1 |
|  | 13c | V | For validation, show a comparison with the development data of the distribution of important variables (demographics, predictors and outcome). | 9/Table 2 |
| Model development | 14a | D | Specify the number of participants and outcome events in each analysis. | - |
|  | 14b | D | If done, report the unadjusted association between each candidate predictor and outcome. | Fig4/5 |
| Model specification | 15a | D | Present the full prediction model to allow predictions for individuals (i.e., all regression coefficients, and model intercept or baseline survival at a given time point). | Fig 4/5 |
|  | 15b | D | Explain how to use the prediction model. | 11 |
| Model performance | 16 | D;V | Report performance measures (with CIs) for the prediction model. | 10-11/Table 3 |
| Model-updating | 17 | V | If done, report the results from any model updating (i.e., model specification, model performance). | - |
| Discussion | | | | |
| Limitations | 18 | D;V | Discuss any limitations of the study (such as nonrepresentative sample, few events per predictor, missing data). | 14 |
| Interpretation | 19a | V | For validation, discuss the results with reference to performance in the development data, and any other validation data. | 12-13 |
|  | 19b | D;V | Give an overall interpretation of the results, considering objectives, limitations, results from similar studies, and other relevant evidence. | 14 |
| Implications | 20 | D;V | Discuss the potential clinical use of the model and implications for future research. | 14-15 |
| Other information | | | | |
| Supplementary information | 21 | D;V | Provide information about the availability of supplementary resources, such as study protocol, Web calculator, and data sets. | S1-S6 |
| Funding | 22 | D;V | Give the source of funding and the role of the funders for the present study. | - |
